# Cost-utility of Protocols of BFM-ALL and UK-ALL for Treatment of Children with Acute Lymphoblastic Leukemia in Iran

**Published:** 2018-03

**Authors:** Hadi HAYATI, Abbas KEBRIAEEZADEH, Mohammad Ali EHSANI, Shekoufeh NIKFAR, Ali AKBARI SARI, Azim MEHRVAR, Elham SHAHGHOLI

**Affiliations:** 1. Faculty of Pharmacy, Lorestan University of Medical Sciences, Khorramabad, Iran; 2. Dept. of Pharmacoeconomics and Pharmaceutical Administration, School of Pharmacy, Tehran University of Medical Sciences, Tehran, Iran; 3. Dept. of Pediatric Hematology and Oncology, Bahrami Hospital, Tehran University of Medical Sciences, Tehran, Iran; 4. Dept. of Health Management and Economics, Tehran University of Medical Sciences, Tehran, Iran; 5. MAHAK Pediatric Cancer Treatment and Research Center, Tehran, Iran

**Keywords:** Cost-utility, Acute lymphocytic leukemia, Children, UK-ALL, BFM-ALL

## Abstract

**Background:**

There is a requirement to assess the effectiveness and resources used in two protocols United Kingdom (UK-ALL) and Berlin-Frankfurt-Munster (BFM-ALL) that are most commonly used to treatment of ALL patients by oncologists in Iran. Accordingly, we analyzed the cost of treatment and utility of children treated with two protocols in Iran.

**Methods:**

The entire medical direct costs of patients in “BFM ALL” protocol and “UK ALL” protocol in multi-centers calculated from Apr 2010 to Jun 2015. For calculating utility and Quality Adjusted Life Year (QALY) of the patients, we used standard questionnaire Health Utilities Index 3 (HUI3). The patients and their parents were interviewed. Data were analyzed using software SPSS18 and EXCEL.

**Results:**

The average direct medical cost for each patient for BFM-ALL was 15026 USD and UK-ALL was 8282 USD which showed a significant difference in the total cost of the treatment in the two protocols (*P*≤0.02). Finally, there was a significant difference in the utility score of the maintenance phase of these two methods (*P*≤0.003).

**Conclusion:**

UK-ALL is dominant and BFM protocol is dominated by both sides total costs and utility and QALY. Mainly, more hospital stay in “BFM ALL” protocol is the cause of raised costs in this protocol. Consequently, by considering different QALYs in the methods and low costs in “UK ALL” protocol, “UK ALL” protocol is more preferred.

## Introduction

Among cancers, childhood acute lymphoblastic leukemia (ALL) has high prevalence (1) but today, ALL has been changed to curable disease due to the medical promotion and its treatment rate has been raised in the recent decades to more than 90% (2). In this regard, there are high costs of cancer in children with ALL in the most of the counties (3). The treatment costs of ALL therapy has been calculated more than 100000 USD in developed countries (4, 5) and in developing countries, high treatment costs prevent many patients with ALL to get right and finished treatment (6). In this context, patients are faced with some problems mostly because of economic leaving (7–9). Some factors greatly rise costs but the length of stay in hospital is a key factor to measure because it is related strongly to long-term treatment and illness severity (5, 10). In economic evaluations, utility scores resulted from the preference-based quality of life tools. The Health Utilities Index (HUI) is the most commonly used among the preference-based measures in pediatric oncology (11). HUI is used to calculate QALY to perform cost-utility analysis (CUA) (12,13). CUA is a helpful method for easier decision making through evaluation of both costs and health outcomes (14). Two protocols, UKALL and BFM-ALL, are most commonly used to treatment of ALL patients by oncologists in Iran.

Berlin-Frankfurt-Munster (BFM) protocols for ALL are in use international and were shown more than 20 yr ago (15) and ALL-UK (United Kingdom) protocol also has led a series of therapeutic trials for acute lymphoblastic leukemia from some decades (16). Oncologists in Iran exert BFMALL and UK-ALL protocols in this scope. Therefore, caused by lack of needed resources and economic assessments, there is a requirement to assess the effectiveness and resources used in the protocols.

Thus, both the costs and benefits of the treatments in the two protocols are surveyed. Therefore, we analyzed the cost of treatment and utility of patients treated with two protocols; UKALL and BFM-ALL protocols.

## Materials and Methods

This study was conducted in 2015 as a retrospective study of the children with ALL referred to pediatric hospitals using based the protocols. From Apr 2010 to Jun 2015 about 250 patients, who had main criteria like finished treatment, no prior relapse, more than 5 yr of age, standard risk ALL, in university Bahrami Hospital and Mahak Charity Hospital were included in the study.

Patients were excluded if diagnosed with infant ALL or were less than 5 yr of age, mature B ALL or high risk ALL, central nervous system (CNS) or testicular involvement. Children who had bone marrow transplantation (BMT) or relapsed were excluded too.

This study has been approved by the code of “IR.TUMS.REC.1394.1988” by the Ethics Committee of the Tehran University of Medical Sciences.

### Outcomes

As mentioned the HUI3 is suggested for measurement of utility and QALY of children more than 5 yr of age by their parents view or for self-assessments by patients more than 12 yr of age (17). Utility scores were based on the direct interview of generic preference of the HUI3 by validated and in-house translated questionnaires. The best-known utility or preference-based measures according to reliability, validity, and feasibility for ALL is the HUI3, there are limitations in concepts and scores of utility measurement tools, but HUI3 for ALL patients could act better than other generic tools (18).

Three treatment phases were defined within each of the BFM and UK protocols. Annual QALYs were calculated, based on mean utility scores and phase durations without discounting because all patients were interviewed in the same year; in 2015 there were different patients over the different phases that were taken the questionnaire, then annual QALYs for each phase by considering the duration were summed to estimate total QALYs for a 5-year analysis period.

### Costs

We used a retrospective approach in data gathered at one point in time. The direct medical cost was calculated for patients in the current clinical practice of disease in 2015. From admission until the end of the treatment, every patient was reviewed and medical costs of each patient were got from their documents. In this period (around 3–5 yr), we checked 10862 discharge sheets of patients treated with the ALL-BFM and ALL-UK protocols. Finally, we calculated the average total direct medical cost for each patient for each finished treatment period. Any costs of bed (hospital stay), visit and consultation, nursing; laboratory tests, radiology, drugs and blood products, laboratory/diagnostic and also visiting specialists, nurses, any hospital admissions were recorded annually. All related costs were calculated using governmental prices and final total costs were adjusted to USD.

Total treatment cost per patient is the sum of total inpatient and total outpatient costs. All costs were adjusted with average annual inflation rates in health care (reported by the Central Bank of Iran-CBI) from 2010 to 2015 (19). Then the average annual exchange rate (US$1. 00=IRR 34000) reported by the CBI was exerted for conversion from Iranian currency (Rials: IRR) into USD to make an easier international comparison (19).

Statistical analyses were completed using excel and SPSS Release 16.0.1 (Chicago, IL, USA). Differences in means were assessed using t-tests.

The Research Ethics Boards of Tehran University of Medical Sciences approved this study. First, each patient was assigned a code then they were informed by our explanations about the study. To confidentiality of patient information, any analysis was based on the codes.

## Results

The prevalence of ALL in boys is more than girls (61% boys–39% girls), in UK-ALL protocol (61% male, 39% female, mean age=8.5±3 yr), in ALLBFM (55% male, 45% female, mean age=9±3.5 yr). And more parents had the nonacademic education (Non-academic=68%; University education=32%) (Table 1).

**Table 1:** Demographic characteristics of patients and parents by treatment protocols for ALL in Iran

***Variable***		***Uk all***	***BFM all***
Number of patients		93	130
Children age (mean ± sd)		8.5±3	9±3.5
Gender children (%)	Male	61	55
	Female	39	45
Education level of parents (%)	Non-academic	68	72
	University education	32	28
Average length of stay (days) (mean±sd)		96± 8	123±11

### Health effects

Patients had lowest mean utility score during the induction phase (UKALL=0.62; BFMALL=0.58) and highest during the maintenance phase (UKALL=0.89; BFM=0.76), for both protocols. The utility scores for the different phases and QALYs were calculated (Table 2).

**Table 2:** Total costs and HRQL scores and QALYs for BFM and UK-ALL treatment protocols (in 2015 US dollars)

	***PHASES***	***BFM***			***UK***			***P-value***
		**Utility scores**	**Duration (year)**	**QALY**	**Utility scores**	**Duration (year)**	**QALY**	
**HRQL**	Induction	0.58	0.08	0.05	0.62	0.08	0.05	0.89
	Consolidation	0.73	0.17	0.12	0.88	0.03	0.03	0.07
	Maintenance	0.76	4.75	3.61	0.89	4.89	4.35	0.003
	QALYs			3.78			4.43	
Average total DMC^*^ per course treatment		15026			8282			0.02
USD-$								

* Direct Medical Cost

Over the 5-yr analytical period, QALYs per patient for BFM (3.99) and for UKALL (3.96) were calculated.

**Fig. 1:**
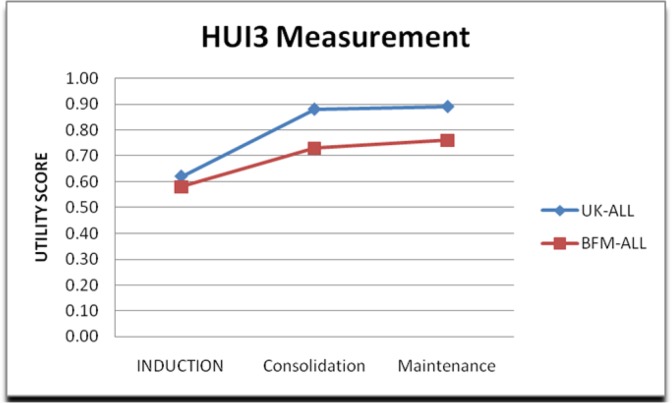
The trend of utility score during the treatment

Table 3 and Fig. 1 show the trend of utility score during whole treatment period that in both protocols the utility level is growing up. Overall, this trend in UK-ALL is upper than BFM-ALL. There is a significant difference in the utility scores of the maintenance phase (*P*≤0.003) of the treatment protocols; it means utility score in UKALL is more than the other. However, the difference during other treatment phases was not significant, (Induction phase (*P*≤0.89) and Consolidation phase (*P*≤0.07) so it means utility scores in these phases somewhat are similar in both protocols and there were no differences in the utility scores.

**Table 3:** The trend of utility score during treatment

***Utility scores***	***UK-ALL***	***BFM-ALL***
Induction	0.62	0.58
Consolidation	0.88	0.73
Maintenance	0.89	0.76

### Treatment costs

In both protocols, the costs of inpatient beds were the most. The average length of hospitalization per patient whole during treatment was 123 d for ALL-BFM protocol and 96 d for UK-ALL protocol (Table 1).

Cost analysis showed that the direct cost per patient, respected the two protocols BFM-ALL was 15026 US dollars and UK-ALL was 8282 US dollars. There was a significant difference in mean total treatment costs per patient between BFM and UKALL (*P*=0.02) protocols (Table 2). There was a big difference between the costs and the cost of UK-ALL is lower than BFM-ALL.

## Discussion

The utility scores of ALL patients during treatment grew up; this result was supported by a study to quantify the health-related quality of life of children with ALL when they were assessed by HUI3 and mean quality of life improved from induction to the post-treatment phase (18). In addition, it is supported to assess quality of life and QALY of children based on BFM-ALL protocol with HUI3 questionnaire that during Induction, Consolidation and Maintenance phases mean HRQL scores were increased too. They were 0.72; 0.78 and 0.85 respectively; also QALYs over 5 yr were 3.99 (20).

Probably its caused by reduced pressure therapy after first phase to patients and more familiar with the disease and treatment procedures after primary confusion and illness severity that at the end of induction phase the disease is somewhat suppressed and patients are placed on the path of recovery.

The BFM and UKALL protocols are not equivalent in terms of health effects a utility and QALY, it may be due to the lack of homogeneity between protocols to treat as duration and intensity and patients treated with the UK-ALL protocol are more satisfied because of less hospital stay and referred less and also earlier recovery comparing with the BFM-ALL protocol.

There is an important result related to a high difference in the mean total direct medical cost between the two protocols, and it is regular that the longer length of hospitalization due to more expenditures and inpatient days between BFM and UKALL lead to differences in the mean total costs of them. Therefore, costs of inpatient beds were the most among other costly factors between both protocols (21).

The mean direct medical cost of patients treated by the UK-ALL protocol was 8282 US dollars. This finding was similar to that in Bangladesh which total treatment cost of children with ALL under a modified UK Medical Research Council XI protocol rose to7672 USD (22).

Cost analysis showed that the average direct cost in BFM-ALL protocol was 15026 US dollars that on this way the mean total costs for BFM in Charlene Rae ‘study in Canada and several European countries were US$ 88 480 (20, 23). Moreover, mean costs from a study in Finland were US $103 250 (5). Children with non-high risk ALL were treated by modified ALLIC BFM2002, the median of hospitalization costs was USD 9900 (10, 21).

Although this is the first study evaluating related costs of treatment and quality of life childhood Acute Lymphoblastic Leukemia in Iran, there are some limitations.

Some parents did not allow us to interview with them due to their emotional state. In addition, some children could not answer the question. In addition, there was not an integral database regarding of costs in the documents.

## Conclusion

The UK-ALL is dominant and BFM protocol is dominated by both sides of total costs and utility. Mainly, more hospital stay in “BFM ALL” protocol is the cause of raised costs in this protocol. Therefore, by considering different utility and QALY in the methods and low costs in “UK ALL” protocol, “UK ALL” protocol is more ‘preferred’. However, as has been said there are some challenges for economic evaluation of cancer in children, and for ALL. In addition, the treatment duration of about 3–5 yr creates many difficulties. Therefore, the final decision-making, by policymakers and physicians, to select one under considerations all clinical and economic conditions is a big challenge. Moreover, each one can make a right decision under their conditions.

## Ethical considerations

Ethical issues (Including plagiarism, informed consent, misconduct, data fabrication and/or falsification, double publication and/or submission, redundancy, etc.) have been completely observed by the authors.
